# Picturing Race in the British National Health Service, 1948-1988

**DOI:** 10.1093/tcbh/hww059

**Published:** 2016-12-12

**Authors:** Roberta Bivins

**Affiliations:** University of Warwick, Coventry, UK

In 1970, Harold Evans, the respected editor of Britain’s best-selling Sunday broadsheet the *Sunday Times* from 1967 to 1981, roundly reproached his fellow journalists for their reporting of ‘race’. Writing for the resolutely middle-brow *The Listener* magazine (published from 1929 to 1991 by the British Broadcasting Corporation [BBC] since 1929 to accompany and amplify the national broadcaster’s educational and cultural mission), Evans asserted that ‘the way race is reported can uniquely affect the reality of the subject itself’. In the matter of race, he observed, the newspapers did far more than fulfil their ‘traditional’ role as a ‘mirror of society’. Instead, ‘stealthily in Britain, the malformed seeds of prejudice have been watered by a rain of false statistics and stories’.^1^ Evans, famously a supporter of US-style campaigning investigative journalism, applied similar techniques to excoriate his fellow journalists. Focusing closely on the language in which stories about non-white migrants and racialized ethnic minorities were reported, he condemned rhetoric portraying migrants as ‘pouring in’ and Britain as being ‘overrun’.

Evans also drew attention to the striking inconsistency with which information about (perceived) race or ethnicity was reported. As the ‘most spectacular’ evidence of such dangerous ‘selectivity’ he pointed to the conservative broadsheet, the *Daily Telegraph*. On 1 March 1969, that paper headlined a coroner’s criticisms of a doctor prominently identified by the *Telegraph* as ‘Pakistani-born’. Yet only inches below, the paper ignored the Asian ethnicity of another doctor (Dr. Hassam Gareeboo) whose heroics saved an infant’s life.^2^

Using another medical story—press coverage of 1961–2 outbreak of smallpox traced to a number of migrants from Pakistan—Evans highlighted the influential role played by press photography in shaping interpretation of the news. Referring to work by the sociologist Eric Butterworth, Evans walked his readers through the exploitation of the outbreaks by an anti-immigration regional newspaper. The *Yorkshire Post*, he claimed, deliberately fostered the impression of ‘a conflict of interest’ between ‘immigrants’ (in fact, British subjects moving entirely legally to the UK from its colonies and Commonwealth) and the local population, not least by portraying a smiling, recently arrived Pakistani migrant next to an unsupported claim that ‘Pakistani smallpox papers can be forged’ to generate the impression that ‘immigrants did not care about the risks to which they exposed local people’.^3^

It is not a coincidence that when Evans turned his attention to the reporting of race in Britain, stories with medical subjects sprang to mind. While the impact of ‘coloured’ immigration on access to housing and employment featured most strongly in popular expressions of discontent, health was not far behind. The advent of the National Health Service (NHS) in 1948 coincided almost exactly with the post-war mass movement to Britain of once-colonial populations from the ‘New Commonwealth’ and thus with the emergence of what became known as the ‘colour problem’. Moreover, concern about the supposed or presumed health impacts of mass migration was strengthened by (sporadic and often self-contradictory) official endorsement.^4^ As Evans asserted in his rebuke, discussions of ‘immigration’—discussions which rarely differentiated between migrating British subjects and immigrating aliens—in post-war Britain routinely employed highly provocative language, accompanied by images intended to inflame. And while racialized migrants were present in large numbers in a variety of industries and were notably visible in public transport services by the 1960s,^5^ it was their work in the NHS that was most frequently visualized in the press. This article argues that in relation to both the NHS and non-white immigration, such visual material often revealed assumptions and expectations that were either too uncomfortable or too thoroughly normalized to be made otherwise explicit.

Evans’ attention to news photography and the suggestive associations created between images and texts on the terrain of the printed page was fleeting if insightful. He devoted far more attention to the rhetoric of race than to its visual representation. Until the last decade, mainstream historical scholarship on race and immigration in post-war Britain has been similarly transfixed by text.^6^ Only with the emergence of a body of scholarship examining the impacts of empire on the British home nations themselves and the growth of interdisciplinary research on visual cultures, has the balance begun to shift towards increasing attention to visual representations, particularly in cinematography and the broadcast media.^7^ Less attention has been paid to the images, photographic and editorial, that accompanied press coverage of ‘race relations’ and immigration, a lacuna which this article will begin to redress. Even in recent innovative work exploring the creation and interpretation of a welfare state that was both ‘post-war’ and ‘post-colonial’, linguistic representations of race, ethnicity, empire and decolonization retain their largely unchallenged centrality.^8^

Moreover, relatively few historians have examined British racial discourses through the lens of specifically medical events, institutions and phenomena.^9^ Yet as Evans’ commentary indicates, such events and sites both prompted and stimulated debates about the very fabric of identity in post-imperial Britain. Here, I will argue that close scrutiny of visual representations of racialized minorities in the NHS can shed new light on British attitudes towards race, ethnicity and belonging in the post-war period. While some images of this intersection are propagandist, candid, opportunistic, or even accidental, particularly in photojournalism and the broadcast media, others are explicitly humorous, ironic, or satirical, most notably a rich seam of editorial cartoons. The latter in particular explicitly use images of racialized bodies and situations in the NHS to comment on and critique social attitudes towards immigration in a welfare state.

This article will use visual representations of white and non-white figures in the context of the NHS to explore perceptions of race and ethnicity, and attitudes towards racism in post-war Britain.^10^ In an initial discussion of the wider British context, it considers the value of editorial cartoons and news photography as tools offering traction on implicit or assumed truths about the NHS. Subsequent sections examine depictions—initially photographic, then in editorial cartoons and for comparison in broadcast and newsreel footage—of non-white nurses, doctors and, largely in absentia, patients. These establish the importance of the NHS as a site in which racial and ethnic inclusion and British diversity could be recognized and portrayed. Little explored by historians, these sources allow consideration of the roles played by both humour and visuality in shaping and expressing responses to human difference understood through the lens of ‘race’. In representing or obscuring racialized figures at work in an institution often regarded as embodying core national values, editorial cartoons and the editorial selection of photographic images deployed visibility itself very deliberately to encode, interpret and challenge attitudes towards such difference in the wider society. The contemporary conflation of skin colour and cultural identity made such work possible, and is correspondingly at the heart of the analysis below. Close reading of specific images also sheds new light on the NHS as an institution imbued with meaning in and for British culture.

## Imagining the NHS: The Value of the Visual

Since 1948, the language of the British NHS has been both possessive and inclusive. Pamphlets, films and other propaganda introduced the service to the population as ‘your’ NHS, and stressed that it was available to ‘everyone—rich or poor, man, woman or child’.^11^ In doing so, documentary sources and political rhetoric reflect and support the ideals of universalism and ‘equalitarianism’ so often associated with the immediately post-war period.^12^

However, if the language of the early NHS encompassed citizens, residents and visitors alike in its remit of care, early press and publicity images of the NHS painted a different picture. Whether produced by the national print media, the Ministry of Works, or the BBC, photographs of the NHS in its early years almost uniformly portrayed white and apparently indigenous British patients, staff and families.^13^ In these pictures, patients and users of the new services visually represented the established ‘vulnerable’ and ‘deserving’ categories familiar to the British public from interwar and wartime campaigns: infants, women, children, workers and increasingly from the mid-1950s, the elderly. Thus visual sources, unlike the contemporary rhetorical and textual evocations of the NHS, very clearly indicate that the possessive community implied by the repeated phrases ‘your NHS’ and ‘our NHS’ was assumed and intended to be a homogenous ‘British public’ that even in 1948 certainly did not exist.^14^

So who was ‘British’ in this period? In law, from 1948 to 1962, British colonial subjects, citizens of its former colonies and Dominions, and those born on the soil of the four ‘Home’ nations shared the status of British subjects, with equal legal rights and privileges.^15^ Contemporary data suggested a British population of approximately 30,000 non-white residents in 1945; historians have estimated the size of this largely settled population at 75,400 by 1951.^16^ Although rarely included in such figures at the time, the diversity of Britain’s wartime and immediately post-war population was greatly increased by the presence of US and colonial servicemen and women.^17^ Between 1948 and 1962, they were joined by some 500,000 primary migrants principally from Poland, the Caribbean and South Asia, but also the Middle East, Africa, and Europe (Irish migrant numbers were not officially recorded).^18^ All migrants from Britain’s colonies and former colonies were entitled to family reunification, and many took up that right. By 1961, London alone reported a population of 242,000 ‘New Commonwealth’ residents.^19^ Yet neither the exotically ‘colonial’ subjects frequently depicted in other contexts, nor Britain’s small but long-established ethnic communities played any significant visible role in official portrayals of the new NHS in its first decade.^20^

Early television programmes and newsreels covering the NHS likewise normally portrayed a service uniformly provided by and to the indigenous majority community. Exceptionally, they might show colonial nurse trainees explicitly preparing to ‘return to their native land [as] skilled professional women’.^21^ However, in December 1958, a BBC documentary broadcast footage of a hospital outpatients department that included an image of a non-white service user as well as a black nurse: a man apparently of African or African Caribbean descent in a crowded waiting room.^22^ Appearing just months after the Nottingham and Notting Hill riots, this matter-of-fact cameo may have reflected official efforts reduce popular racism through a programme of public education.^23^ Only a year later, the ground-breaking medical soap opera *Emergency Ward 10* introduced a black nurse, played by Gloria Simpson, who became the first black actor to be cast in a UK soap opera; by 1961, it would include its first black doctor, the recurring character Dr Jeremiah Sanders (played by Clifton Jones).^24^ And indeed by the late 1950s, perhaps it was no longer possible either to ignore or to obscure—visually or rhetorically—events and trends both within and beyond the NHS that challenged homogenous visions of Britishness, disrupting expectations about health citizenship and ‘earned’ entitlement to state-funded medical services. New patterns of migration, new types of labour, new sites of interaction, and new nodes of tension between recent arrivals and established residents combined to spotlight the visibility of Britain’s increasing human diversity in the flagship service of the Welfare State.

Understood symbolically as the starting point of mass migration from Britain’s colonies and the nations of the ‘New Commonwealth’, the ship Empire Windrush disembarked its passengers, including 494 West Indians and 65 Poles at Tilbury docks on 22 June 1948, only weeks before the NHS Appointed Day.^25^ As British subjects, the former were immediately eligible to receive all NHS services. The latter, who might have been excluded as European ‘aliens’, gained their own entitlement under the Polish Resettlement Act of 1947. They joined a swelling but largely unquantified population of Irish migrants and seasonal workers; medically screened, selected and counted ‘European Voluntary Workers’; elite African and South Asian students; warmly welcomed migrants and visitors from the Old Commonwealth and United States; and independent economic migrants and refugees from across the expansive historical footprint of the already-shrinking British empire.^26^

Subjects or aliens, visitors or settlers, these newcomers prompted curiosity, humour (both good natured and hostile), and fear. In relation to the NHS, they often triggered rhetorical displays of anxiety and angry possessiveness. Debates about the ‘abuse’ of the NHS by individuals entering the UK specifically to avail themselves of its new health services began within the first year of the Health Service.^27^ By the 1950s, accusations that immigrants in particular burdened and exploited the NHS were commonplace. Yet paradoxically, some of the in-comers also became symbols of care and cure, of the fragility of the nascent Service itself, and of its dependence on imported labour. These two tropes—one depicting burdensome and undeserving immigrants; and the other focused on visibly ‘foreign’ doctors and nurses—dominate representations of the intersection between post-war migration and the NHS from its earliest years. They also lay bare marked differences between visual and textual discourses of racialized migration in this context. Scholars of migration, almost irrespective of time and place, have documented the familiar view of the migrant as a vector of disease, and post-war Britain was no exception.^28^ However, while the infectious and scrounging immigrant was omnipresent in debate, this stereotype was rarely represented in images. In contrast, the migrant saviours of the NHS were potently and prolifically visualized, particularly in news photography and editorial cartoons. Two factors help to explain both the former absence and the contrasting abundance of imagery documenting West Indian, African and Asian NHS workers. First, as epidemiological data have consistently confirmed, most migrants were (and are) in fact healthy young adults. Thus, except in relation to maternity services, they made little use of the most dramatic and photogenic NHS services, the hospitals. Second, from the 1958 Notting Hill and Nottingham riots through to the 1978 General Election, fears about Britain’s growing racial diversity were matched by wide-spread anxiety about the emergence and potential ill-effects of popular racism. At least in relation to the NHS, evidence suggests that the latter inhibited the creation of factually inaccurate images showing immigrants ‘battening’ on its services, while stimulating the production of visual representations that critiqued such explicitly racist claims by accurately depicting their significant labour contributions.

## Becoming Visible: Incorporating ‘Colour’ into Visons of the NHS

As commentators across the political spectrum agreed, ‘colour’ made some migrants especially identifiable. Again and again, social investigators specifically targeted their ‘visibility’ itself as both novel and damaging, either to individual migrants or to the wider host community.^29^ By 1955, sociologist Anthony Richmond had identified this visual trait as one of particular salience to social mobility: skin colour became ‘the outward and visible sign’ of migrants’ low status as an ‘out-group’, and one that was intractable precisely because of its ‘permanence’ as compared to more evanescent markers including ‘wealth, education…dress or uniform, and the performance of ritual patterns of behaviour’.^30^ In the early 1960s, social anthropologist Sheila Patterson observed British antipathy to the ‘visible alienness’ of the newcomers, while Ruth Glass asserted that their visible ‘distinctiveness’ could prompt both ‘antagonism’ and ‘ardent sympathy’.^31^

These scholarly observations both filtered into and reflected wider discourse around race in Britain. In 1956, for example, the author of a study of ‘coloured workers’ in the Birmingham area mused that ‘[f]rom the point of view of numbers’, the problem was ‘not a big one’. Instead, ‘part of the trouble is that everyone can see that a Jamaican is from overseas, whereas this is not so obvious in the case of an Irishman, an Australian, or even an Italian’.^32^ He explicitly cited Richmond’s frame of the ‘highly visible minority’ in support of his claim.^33^ The idea that physical visibility could explain the emergence and persistent of racial antipathies and tensions rooted in ‘an emotional and genetic chaos to which the majority of mankind is not inured’ became the common sense of the period.^34^ It permeated discussions of British xenophobia, and was naturalized by indigenes and immigrants alike, at least in press reporting.^35^ It is not a coincidence that the term most frequently used to describe unwelcome migrants from Britain’s remaining tropical colonies and growing New Commonwealth from the 1940s to the 1970s was ‘coloured’, nor that the terms ‘immigrant’ and ‘coloured’ became essentially synonymous in both political and common speech. As one parliamentarian observed, the debate over immigration had become one about the ‘colour problem’ precisely because ‘the coloured immigrants can so easily be distinguished’.^36^ The intense attention paid to the visual marker of skin pigmentation notwithstanding, ‘colour’ certainly did not exhaust the perceived distinctiveness of New Commonwealth migrants. Rather it served as a synecdoche for the array of physical, cultural and linguistic differences that distinguished these populations.

Indeed, it is in part this focus on the differential visibility of migrants from Britain’s remaining tropical colonies and lost empire that makes imagery a useful window on the articulation and understandings of racialized human differences, later often euphemized as ‘ethnicity’, in explicitly universal and inclusionary institutions like the NHS.^37^ Here, editorial cartoons and humour in particular are valuable historical sources, since their power to provoke and amuse relates directly to the skill with which their creators ‘encapsulate the public mood’ and tap their audience’s sense of irony, incongruity, and even moral superiority.^38^ This latter factor is especially evident in cartoons addressing race and race relations in the post-war period. Angela Rosenthal and Adrian Randolph have observed the importance of humour as ‘a valuable strategy through which highly sensitive and difficult issues could be raised for “serious” consideration’. In anxiously post-colonial Cold War Britain, few issues were either as sensitive or as difficult as race. Editorial cartoons and other forms of visual humour could challenge social norms, but usually did so in complicity with their viewers.^39^ Writing in 1966, the prolific editorial cartoonist David Langdon observed that ‘haranguing’ or ‘politically committed’ cartoonists were scarce in the post-war period, overtaken by the novel televisual ‘satirical revues’ (including the BBC’s ‘That Was the Week That Was’), but also by public taste.^40^ Moreover, as Stuart Hall argued, the ‘comic register’ protected audiences from ‘acknowledging their incipient racism’ while recognizing it as a social problem.^41^

Attention to images in this medical context also respects the heightened importance of visual evidence as a crucial source of diagnostic authority in the health services. For example, in relation to tuberculosis, the archetypal ‘immigrant illness’ of the post-war period, visual evidence of health or disease in the form of chest x-rays was deemed essential. For decades, the British Medical Association campaigned vigorously and vocally for the medical screening of migrants either before departure to the UK or on entry at British ports.^42^ Their calls uniquely privileged radiographic examination, the one diagnostic technique that produced visual (if neither particularly reliable nor unambiguous) evidence of pulmonary tuberculosis. Moreover, medical responses to non-white immigration and ethnic communities frequently emphasized their status as visible and ‘easily recognized’ targets for surveillance or intervention, while reportage on these groups was also highly likely to include visually striking graphics charting the epidemiological or physiological distinctiveness of the migrants and their descendants.^43^

Yet beyond the professional journals, with their unique access to and perspective on the immigrant as a patient and an epidemiological variable, visualizations of the infectious immigrant remained largely imaginative and rhetorical. Very few photographs of non-white patients appeared in the factual media between the 1950s and the 1990s, even as the population of potential subjects expanded rapidly. In part, this surely reflects changes in the practices of public health under the NHS regime of universal access. The large scale and highly visible public health interventions of the interwar and early post-war years, including the mass miniature radiography campaigns that positioned x-rays as the diagnostic gold standard in the eyes of the public, were gradually replaced by individualized preventive strategies operated through the private doctor–patient encounter. Press photographers and broadcast journalists had limited access to these private moments, which were, in any case, unlikely to provide the drama or controversy that would have justified their use.^44^ It is indicative of both editorial biases related to the ‘infectious immigrants’ trope, and the rarity of opportunities to represent it photographically that when experimental radiographic screening facilities were introduced in 1964 to check migrants for tuberculosis at Heathrow Airport, they were the subject of considerable media coverage, including a series of photographs in the anti-immigration national newspaper, the *Daily Mail*.^45^ Yet even these, following Pakistani work permit holder Abdul Haq through the health checks as he entered Britain for the first time, could not show the examination itself. That encounter was private, despite being, in the words of the reporter, the crucial final step of the process that ‘made an Englishman’.^46^ Notably, no official publicity photos were provided of these politically sensitive facilities by the Ministry of Health, despite the anticipated popularity of the innovation with both doctors and the public.^47^

Editorial cartoons were not hampered by similar constraints; cartoonists could draw any situation, including those from which the press was excluded. Nor did editorial cartoons require particularly visible, striking and familiar markers of disease to make their point; in the nineteenth century, when the ‘infectious immigrant’ *was* frequently visualized, figures were often simply labelled as the diseases they were intended to represent.^48^ And yet, strikingly, post-war editorial cartoonists generally did not adopt this provocative theme, or seek to portray a non-white population burdening the NHS. Indeed, among the 755 digitized editorial cartoons depicting either explicitly or apparently NHS patients as subjects in the British Cartoon Archive (one of the largest repositories of editorial cartoons and cartoonists’ papers in Britain, with images drawn from a broad political spectrum of the national news press), only fifteen published between 1948 and 2015 included non-white figures; of these, six were published after 2010.^49^ Even this handful of mid- and late-twentieth-century images generally deployed non-white patients as visual placeholders for political issues: for example, one 1965 Cummings cartoon for the *Daily Express* used the figure of a large black man waiting in a general practitioner (GP)’s surgery to represent the political problem of ‘immigration’ itself rather than any particular medical burden.^50^ The satirical magazine *Punch*, too, painted the users of the NHS as white; only one *Punch* cartoon on a medical subject published after 1948 showed a dark-skinned patient.

In fact, research thus far has uncovered only one editorial cartoon in the national press that directly connects imported disease and immigration. Published at the height of the 1961–2 debates over the Commonwealth Immigrants Act and at the peak of the simultaneous imported smallpox outbreaks, it explicitly critiques government attitudes and policy, rather than the migrants themselves. In the foreground, a cage of dogs destined for the required 6 months of quarantine on entry to the UK is guarded by the unfortunate RAB Butler, who was forced by his role as Home Secretary to preside over the passage of legislation he clearly viewed as an unpleasant necessity.^51^ A speech bubble rising from the cage grumbles ‘So it’s alright to let *humans* through—*they* may only have smallpox!’ In the background, a stream of Asian men march out of an aeroplane labelled ‘from Pakistan’, to flow unimpeded into Britain.^52^ While newspapers frequently depicted rail stations, ports and airports crowded with arriving ‘immigrants’, sometimes—as Evans noted in 1965—in conjunction with news stories about imported disease, they did not (perhaps could not) portray similar crowds awaiting medical attention. Even during the smallpox outbreak itself, Pathé footage and news photography of sometimes rowdy vaccination queues include no dark faces, probably because such incomers were vaccinated before or upon arrival in the UK.^53^ Britain may have been ‘a magnet of attraction’ precisely because of its ‘superior … social services’, and new arrivals were certainly suspected of participating in ‘an organized racket to enjoy the benefits of the welfare state’, but these putative attractions produced only one visible and regularly visualized effect for the NHS: an increasing and optically striking diversity among its staff.^54^

## ‘Without Them, the Health Service Would by Now Have Collapsed’: Picturing Medical Migrants

Photographic archives are replete with images of the NHS, both published and unpublished.^55^ Among the most popular subjects for published images were hospital nurses—and in these photos, the rich ethnic diversity of NHS staff is evident from at least the 1950s. In ethnically mixed groups, among their non-white peers, or working alone, smiling black women in white were highly visible in news photography. One *Daily Herald* article, published in 1955, made a specific feature of their skin colour and their smiles. ‘These coloured nurses are happy’, the headline proclaimed.^56^ A caption above two photos (one of a black nurse and her white colleague talking to a reclining white female patient, and one of a black nurse alone, gently resting one hand on the shoulder of a gratefully smiling white woman, while balancing a medicine cup and saucer in the other) proclaimed: ‘Efficient, dignified—That’s what hospitals say’. The article opened with a strong claim, both supporting and drawing on these images: ‘Nearly 2,000 coloured nurses work at 300 hospitals in England and Wales, but only once—at Swansea a few days ago—have white nurses objected to them. The reason is simple. Coloured girls make as good nurses as white, and sympathy and kindness—like sickness—know no colour-bar’. Quotes from the nurses themselves, all from Queen Mary’s Hospital in Kent, close to London, offered an equally cheerful perspective, rejecting the existence of racial prejudice inside the NHS, even when they admitted experiencing its effects beyond its wards. It is worth noting that both of the student nurses are also quoted revealing plans to return ‘home’ after completing their training.

Consistently, Britain’s national papers, even those like the *Daily Mail* which were resolutely hostile to immigration in general, represented black and Asian nurses either in a positive light, or simply as an expected part of the NHS context. For instance, although doctors and nurses feature both in the text and in the illustrations of the provocative ‘The Dark Million’ series of articles in the *Times* in 1965, it was an immigrant Nigerian nurse, lovingly cradling a newborn baby that illustrated an article claiming ‘Life would be harder for all of us without coloured labour’.^57^ Tellingly, like the *Daily Herald* article above, this *Times* account took pains to depict the NHS as free from racial prejudice. Thus, while the article documented the unpleasant racism that faced ‘coloured’ workers in industry, its author comfortably claimed that similar conditions did not apply in the NHS, where ‘[c]oloured professional people’ were ‘performing an obviously useful service’—and were not ‘obviously competing for jobs’.

By the mid- to late-1960s, non-white nurses mingle with their white counterparts freely on pages of the national newspapers, as in photos of a London service commemorating the death of nurse Edith Cavell and a royal awards ceremony (though none are included in the recurring images of nurses modelling new uniforms until 1971).^58^ Indeed, a rare photo of non-white patients in this period depicted two black nurses, hospitalized along with two of their white colleagues by the flu epidemic they had previously been treating.^59^ Their presence in the NHS has become unremarkable; arguably, even when black and Asian nurses appear in news photography, they are no longer ‘visible’ as outsiders. For example, a *Times* article assessing the NHS on its 30th anniversary depicted a white-uniformed black nurse manipulating high-tech equipment above a supine white woman; neither her race nor race in general was an implied or explicit subject in the article itself.^60^ A critical 1986 article in the *Daily Mail*, while portraying ‘immigrant women’ and their pregnancies as an unanticipated burden on the NHS, was nonetheless illustrated by stock photograph of a ward staffed by a non-white nurse.^61^ A similar image accompanied a 1998 expose, ‘How the NHS Betrayed my Mum’ that railed, inter alia, against ‘an immigrant’ who, the outraged author claimed, received better NHS treatment.^62^

## Drawing the Sting of Racism: Editorial Depictions of NHS Diversity

While photojournalists had comparatively limited scope to portray many aspects of the NHS, editorial cartoonists enthusiastically seized opportunities to depict the NHS and its staff. The British Cartoon Archives has digitized hundreds of editorial cartoons published or drawn between 1948 and 2015 in which doctors (997) or nurses (505) are indexed subjects.^63^ These professional groups also appear frequently in editorial cartoons with non-medical topics. In part, of course, their popularity stems from the ready recognizability and familiar connotations of medical uniforms and environments. The nurse (or the nurse’s uniform) in particular was a synecdoche for care, for devotion, and at times for the NHS itself—but also, especially in relation to ‘Matron’, for petty tyrannies, finger-wagging conservatism and a hidebound insistence on nonsensical or poorly understood rules. Even relatively minor news stories like Winston Churchill’s brief 1962 stay in hospital for a broken thigh prompted no fewer than six editorial cartoons set on NHS wards (the majority commenting on hospital diets and rules about smoking). Similarly, Ringo Starr’s tonsillectomy in 1964 occasioned at least four cartoons, mostly re-siting Beatlemania to the wards.

**Figure 1:**
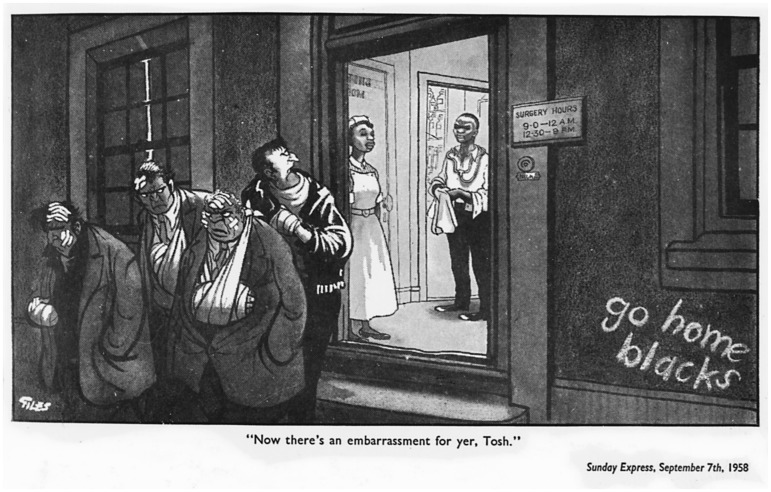
Giles (Ronald Carl Giles), ‘Now there's an embarrassment for yer, Tosh’, Sunday Express 7 September 1958, © N&S Syndication, British Cartoon Archive.

Larger political events, too, were interpreted through the lens of the NHS, including matters of the Budget, NHS funding, and the changing status and provisions of Britain’s Welfare State. In particular, cartoons addressed the evolving post-war debate about race relations in Britain through the lens of the health services with noticeable frequency. The Nottingham and Notting Hill riots in the 1950s, the rise of Powellism in the late 1960s, and the ‘race riots’ of the early 1980s all prompted both rhetorical imagery and editorial cartoons reflecting the centrality of racialized medical staff to the continued functioning of the health services. Unlike the ‘infectious immigrant’, this trope is commonly represented, both rhetorically and visually, through irony. Crucially, such humour generally includes its audience among the knowing, and points to others—here, racists and xenophobes—as the butt of the joke. Thus, in 1958, an early visual representation of non-white NHS staff tackles the racism that sparked the Notting Hill Riots head on (Fig. 1). Cartoonist Giles (Ronald Carl Giles), known for his emphasis on social rather than political commentary and a committed anti-racist, portrayed four thuggish and heavily bandaged white men walking grumpily into the darkness from a brightly lit doctor’s surgery.^64^ Its posted opening hours are Stakhanovite: 9:00–12:00 am and 12:30–9:00 pm; scrawled beneath them, graffiti reads, ‘go home blacks’. Standing in the doorway are a black doctor and nurse. The nurse is serene, but the shirt-sleeved doctor’s expression is irritated, perhaps even disdainful as he wipes his hands on a clean towel. The caption grumbles ‘Now there's an embarrassment for yer, Tosh’.^65^ The message is clear: racism and racial violence were shameful, and were made more so by the fact that racial minorities were so clearly the mainstay of the NHS, providing treatment even to those who abused or sought to exclude them.

**Figure 2:**
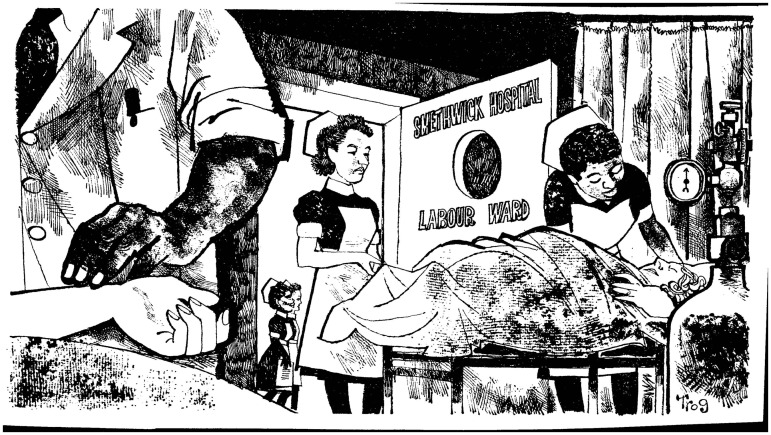
Trog (Wally Fawkes), uncaptioned cartoon, New Statesman, 20 November 1964, © Wally Fawkes, British Cartoon Archive.

Editorial cartoons similarly exploiting the highly visible presence of ‘immigrant’ doctors and nurses (and other staff) in the NHS became a common response to the milestone events in race relations and British racism for the remainder of the twentieth century. They marked debates around the Commonwealth Immigrants Act in 1962, hostile reactions to the various Race Relations Acts, Britain’s 1968 exclusion of Kenyan Asian refugees, Powellism, and even smaller news stories that revealed racism in the NHS itself.^66^ For instance, cartoonist Trog (Wally Fawkes) contributed a striking image to the liberal *New Statesman* magazine, published not long after the infamously race-dominated Smethwick by-election. Here, two white women lie in a darkened hospital labour ward staffed entirely by dark-skinned nurses and doctors, while a pair of nurses, one black and the other white, hurry past in the corridor beyond (Fig. 2).^67^ The cartoon’s obvious reference to Labour’s recent loss in the racially charged Smethwick election campaign would have been as clear to viewers as the irony of its immediate prompt, the local Labour Party’s subsequent refusal to allow its defeated candidate to hold a racially mixed gathering in the Labour Club.

### Naturalizing Diversity: Racialized Nurses and the NHS

Perhaps even more interesting and revealing, however, are the ways in which non-white staff, and especially nurses, populate editorial cartoons about the NHS which are not specifically addressing questions of race relations or immigration. From the 1960s, cartoons tackling debates over the NHS, its funding, its failures, and the activism of its staff increasingly included depictions of black (and less frequently, Asian) nurses. No longer the specific subjects of the cartoonists’ humour, these women—this research has found no examples of male nurses of any ethnicity—were incorporated into the popular visions and expectations of the NHS upon which editorial cartoons drew for their verisimilitude. That is, they provide the conventional setting from or within which the political message or ironic claim becomes distinct and legible. And these depictions are, indeed, highly conventional; non-white nurses comply with exactly the same stereotypes applied to their white counterparts. Whether young and buxom or middle-aged and severe, they are appurtenances of the ward, the waiting room and the NHS and are only occasionally to be found outside its corridors. In fact, ‘Commonwealth’ migrants filled 30 per cent of pupil nurse vacancies and 29 per cent of pupil midwife slots by 1968, and thus represented a very significant proportion of the hospital nursing labour force. Their regular inclusion in editorial cartoons was, at the very least, an accurate reflection of the NHS ward environment.^68^

**Figure 3:**
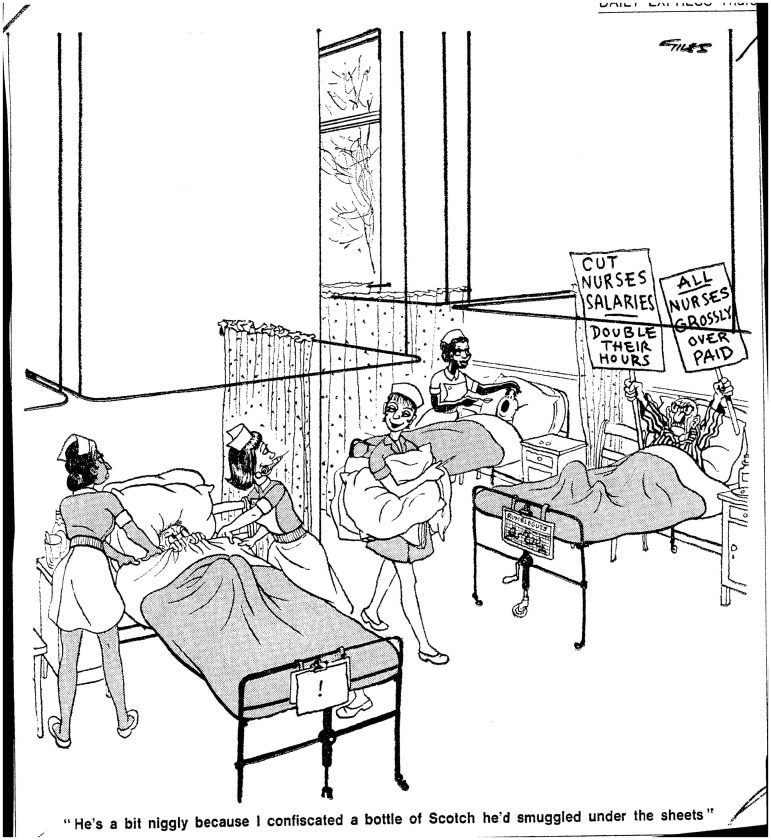
Giles (Ronald Carl Giles), ‘He's a bit niggly because I confiscated a bottle of Scotch he'd smuggled under the sheets’, Daily Express, 25 February 1971, © N&S Syndication, British Cartoon Archive.

From 1948 onwards, cartoons also presented the nurse as a political actor, striking, protesting pay or working conditions, or refusing her usual caring services; here too non-white nurses marched alongside their white colleagues. Giles, drawing for the *Daily* and *Sunday Express* papers from 1948 to 1989, was among the most prolific cartoonists of nursing and ward life, set implicitly or explicitly in the NHS; his famous ‘Giles Family’ series in particular included frequent visits to the wards. From 1962, these cartoons routinely included black and Asian nurses. Giles visually denoted their ethnicity by stereotypically racialized features (prominent lips, hairstyles, eye-shape, and sometimes additional skin stippling)—but their activities and roles were identical to those performed by the white nurses depicted working alongside them (Fig. 3). These women were not the subject of his cartoons, which generally poked fun at generically British social conventions (including idealized visions of nurses themselves as ‘angels of mercy’). Instead their presence was woven into the fabric of NHS life. Like the rest of his highly detailed background drawings—half-plucked grape stems, rowdy children, and endless cups of tea, they reflected the service as it was, or was assumed to be. It is noticeable, however, that Giles’ racially marked nurses rarely speak, nor has this research discovered any ethnically marked ward matrons in his images.^69^

Jak (Raymond Jackson, an editorial cartoonist for the *Evening Standard* known for his strong right wing views) also silently integrated his hospital scenes from the early 1960s, while Mac (Stan McMurtry), drawing for the *Daily Sketch* and then the politically conservative *Daily Mail* followed a similar visual strategy from 1969.^70^ Perhaps Jak was making a political point as well in a 1964 cartoon—also in the wake of the Smethwick scandal—with a noticeably high proportion of black nurses, but its principal subject was Ringo Starr’s tonsillectomy and the already ludicrous market for Beatles memorabilia.^71^ However, his approach to representing race in the NHS offered none of the explicit conservative political critique that characterized his other cartoons.

Strikingly, such editorial depictions of visibly racialized nurses are without exception either positive, generally when explicitly reproving or countering racism or, as discussed above, matter-of-fact. Few if any editorial cartoons in the mainstream press specifically portray non-white nurses critically, though, like their white counterparts, they are often sexualized or made the objects of sexual approaches or humour by patients or doctors. Not until 2013, in a glut of editorial cartoons responding to scandalous failures in care at the Mid-Staffordshire (NHS) Hospital, have I found an editorial cartoon in which a racialized nurse was represented critically. Even in this case, a cartoon drawn by Mac for the *Daily Mail* and using the trope of the three wise monkeys, race is not the principal subject. Rather, the racialized nurse figure operates as a stand-in for *all* Mid-Staffordshire nurses, just as its two white male figures—a traditionally white-coated doctor, and a plump and be-suited manager—represent the other hospital professionals who ignored the hospital’s rising death rates (represented by hearses passing behind its three seated figures).^72^

### White Coats and Witchdoctors: Racialized Doctors and the NHS

Visibly ethnic doctors, in contrast, prompted more ambivalent imagery. Like nurses, black and Asian doctors were often represented in cartoons explicitly addressing the bitter ironies of British racism, given the essential role they played in the NHS. Enoch Powell’s over-heated anti-immigration rhetoric in particular prompted such responses in the late 1960s; Stanley Franklin, drawing for the *Daily Mirror*, offered a fairly typical visual rebuttal of popular racism with an image of an explicitly NHS hospital corridor occupied by a serious Asian doctor reading a chart while two black nurses attentively move a patient to the operating theatre. In the foreground, two white figures look on. A man in a flat cap (commonly used to denote exactly the white working class audience most visibly captured by Powellism) ask: ‘Is this some of the “Alien-occupied territory” Powell’s talking about?’.^73^ Here at least some of the cartoon’s impact surely derives from the irony of such a familiar and inclusive national institution being described by its traditional beneficiaries as ‘alien’ territory. Drawing for the middle-class audience of *Punch* magazine, David Langdon made a similar point in 1969. As he is wheeled into an operating room staffed entirely by black figures in surgical whites, a walrus-moustached white male patient anxiously addresses his black doctor: ‘I beg you to excuse the release of any deep-seated prejudices while I’m going under’. Behind the pair, a black nurse passes by unconcernedly. Langdon’s vision is one of an NHS entirely and routinely staffed by ethnic minorities, and therefore one in which the hierarchies of race and class that were so ‘deep-seated’ in Middle England were themselves humorously reversed.^74^

**Figure 4:**
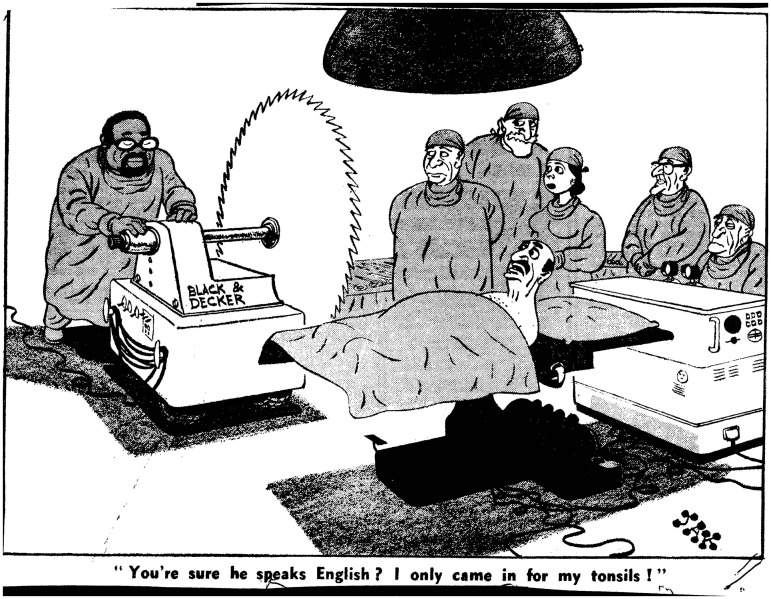
Jak, ‘You’re sure he speaks English? I only came in for my tonsils!’, London Evening Standard, 21 February 1969, © SOLO Syndication, British Cartoon Archive.

However, it took little prompting to draw out far more negative images of ‘dark doctors’. For instance, in 1969 an anonymous senior surgical consultant’s critiques about ‘immigrants’ medical training not only made the front page of the *Times*, but generated a host of very negative cartoons.^75^ In the *Evening Standard* cartoon, Jak depicted a large black surgeon pushing an even larger circular saw up to the operating table, which a crowd of white medical staff look on dubiously. The anxious patient supplied the caption: ‘You’re sure he speaks English? I only came in for my tonsils!’ (Fig. 4).^76^ The *Daily Mail* offered an Emmwood effort depicting a turbaned snake-charmer performing in front of a sign advertising ‘Dr. Babu Anti-Snake bite serum. Englisch Badli Spoken’. Beneath, in case the reader missed the visual ‘joke’, the paper added a caption in doggrel: ‘Doctor Babu from Bombay,/Very good doctor in every way,/Came to London, National Health,/Plenty patients,/Plenty wealth,/Oh my goodness! Gives wrong unction,/Very soon back in Bombay Junction’.^77^ In contrast, on the same day David Meyers, also in the *Evening Standard*, portrayed a (white) consultant reassuring Matron on the ward round, while a monstrous (white) doctor strangles a patient in the background: ‘Our immigrant doctors I find splendid—but I am a bit worried by Jekyll’.^78^

Two other cartoonists seized the opportunity to make ‘witch-doctor’ jokes, illustrated with the broadest of African racial stereotypes.^79^ Similar tropes had appeared earlier in the decade in relation to stories about doctor shortages in the NHS.^80^ By 1971, however, the witchdoctor imagery was turned on its head, to rebuke Enoch Powell’s racist demagoguery. As Powell cowers in his bed, a black witch doctor addresses a stereotypical female ‘native’ (complete with nose-ring and bone hair pin): ‘Two tablets every day, Mrs. Powell—these hallucinations of “black bogeymen everywhere” should soon pass’.^81^ In 2001, cartoons responding to yet another news story about NHS recruitment of overseas doctors to plug domestic gaps again turned to the visual metaphor of the witchdoctor. By this point, however, such highly racialized imagery was no longer acceptable even within the elastic frame of the editorial cartoon. The British Medical Association protested to the editors of both the *Daily Mail* and the *Daily Star*, which printed the offending cartoons, noting that ‘attempts at humour of this kind perpetrate a climate of intolerance that breeds racism’. While the *Daily Star* refused to back down, *Daily Mail* editor Paul Dacre apologized. The *Daily Mail*, he stated, had no wish to ‘give rise to racism within the wider community of healthcare’.^82^

As well as attracting more negative editorial portrayals, non-white doctors were less likely to appear simply as part of the NHS mis-en-scene. While Jak depicted without comment an ethnically mixed group of doctors marching out to protest for more pay in 1970, and Giles included them occasionally from 1973, such apolitical representations remained rare.^83^ Where the image of the non-white nurse had lost any hint of political or racial salience by the late 1960s, after a decade of ever-growing anxieties about both ‘race’ and ‘immigration’, portrayals of black and Asian doctors still courted controversy. Comparing editorial depictions of ethnically marked nurses and doctors, it seems likely that the ease with which black and Asian nurses could be understood as fulfilling a traditional ‘service’ role was one factor in rendering their professional activities in the NHS neutral and invisible. Unlike black and Asian doctors, their presence on the wards was less disruptive to established hierarchies, at least for middle-class patients and observers (though for working class patients, their authority as well as their racial difference could be a threat).^84^

Cartoonists, of course, could choose to include or obscure the very extensive presence of non-white doctors in the NHS; newspapers could print or archive photographs of these disruptive figures. As the *Illustrated London News* reported in 1968, such doctors made up a very considerable percentage of hospital staff: citing the British Medical Association, it announced that 46 per cent of hospital doctors below the rank of consultant, 46 per cent of registrars, and 59 per cent of senior house officers already came from abroad.^85^ It is therefore especially revealing that this generally pro-immigrant article was itself accompanied by an image of a black nurse and a *white* doctor at a patient’s bedside.

In contrast, by the end of the 1960s, factual films and documentaries could only erase non-white hospital doctors by very skilful editing or set management. The response of a 1968 television documentary indicates that the intransigently visible presence of non-white doctors (and patients) in the NHS remained problematic in actuality footage, however normal it had become in print by the end of the 1960s. In this critical BBC documentary ‘celebrating’ the twentieth anniversary of the NHS (and gloomily entitled ‘Something for Nothing’), a young Indian doctor is disparagingly described as ‘a stop-gap’ to address the system’s failure to retain dissatisfied clinicians and consultants fleeing the NHS for more hospitable climes abroad: ‘He comes from India’, the narrator proclaims. ‘On May the third, he arrived in England with three pounds ten … took a room at an Indian students’ hostel in London, went on National Assistance, and began writing applications for jobs’.^86^ The documentary later portrays his medical peers, young doctors (and a handful of nurses) from India and Pakistan, as populating a dystopic Victorian hospital environment—the exact opposite of the modernity so confidently predicted for the NHS in earlier decades. In the same film, the camera scanning a row of patients waiting in a hospital emergency room twice cuts away from women of Asian descent. It lingers instead on the familiar faces of the indigenous elderly and young.^87^ Another visibly ethnic patient flashes across the screen only briefly and almost out of frame.^88^ Broadcast just months after Enoch Powell’s catalytic ‘Rivers of Blood’ speech, perhaps the documentary’s producers felt that images of ethnic patients ‘burdening’ the NHS represented too great a provocation, even for a film clearly intended to provoke.

Its controversy-courting focus on migrant staff clearly caught the viewers’ eyes. A *Daily Mail* review of the ‘highly watchable and emotional’ programme described its depiction of the NHS as dwelling on ‘the grim side’, but breezed over the ‘well-worn facts’ about shortages of doctors, nurses and cash. Instead, the reviewer highlighted ‘some new shockers, like our almost complete dependence on immigrant doctors’.^89^

It is in that final line that the difference between rhetorical, editorial, photographic and documentary imagery about race in the NHS really becomes evident. A wealth of news stories and commentary pieces across the lifespan of the NHS exist to document the well-established and still very current trope of immigrant doctors and nurses as the saviours of the NHS. In a 1970 commentary on his radio documentary exploring responses to immigration in the depressed northern industrial town of Blackburn, reporter Jeremy Seabrook offered his own perception of how this vision shaped popular discourse about race:

Opinions [on race] are formulated on an *ad hoc* basis arising out of the circumstances of each conversation. Most people are subconsciously aware of certain prevailing attitudes towards the coloured population … which can be dredged forth as necessary. Many of these attitudes are encapsulated in current received ideas and set phrases like ‘if it weren’t for the immigrants, the Health Service would collapse’, or ‘How would you like your daughter to marry one of them?’. And these are duly given utterance at the relevant point in any discussion.^90^

Editorial cartoons reflected this now-familiar vision back to readers, most commonly through the lens of humour. They allowed their audiences to distance themselves from the discomforting evidence of wide-spread racial prejudice, but perhaps also from the degree to which the editorial vision of a dark-skinned NHS reflected as well as exaggerated the realities of staffing the Service. News photography depicting the NHS, constrained by the limits both of the print format and of medical ethics, most often served to reinforce and amplify than to alter existing images of the NHS. It was the apparently candid television footage capturing and exposing, without the buffer of humour, a highly ‘coloured’ service, rather than column inches describing it, that retained the power to shock. The heightened but entirely recognizable reality made visible in this format deliberately stripped away the deadening familiarity of the rhetorical imagery, and erased the protective distance created by the audience’s inclusion in the knowing humour of the editorial cartoonists.

## Conclusions

Across the visual formats I have discussed here, one shared feature emerges: the NHS context in each case operated to encourage and indeed mandate interracial encounters. It both was, and was regularly depicted as the archetypal space where Middle England met migrant England. Indeed, it is possible to argue that the NHS played a very specific role in the visual culture of human difference in Britain in this period. Photographically, the NHS offered a space in which the imaginative vision of Britain as a ‘tolerant nation’ could be physically as well as rhetorically represented. In editorial cartoons, in contrast, the NHS provided a space of (especially racial) saturnalia, both in terms of its social inclusivity and by reversing the expected polarities of the era’s perceived and expected racial or ethnic hierarchies.^91^ Boundaries and expectations were perennially transgressed in this body of work, to the point that such transgressions themselves became the genre’s norm, as in the many images which depicted the NHS as a space in which chagrined racists inevitably and embarrassingly encountered racialized medical professionals. And yet both news photography and editorial cartoons depicting ‘race’ in the NHS ultimately served the same agenda: they reinforced the normative vision of British culture as fundamentally free from racial bias, whether by depicting inclusivity, or by mocking racism. That the NHS was the site within which representations of this vision were seen as credible (despite considerable evidence to the contrary) speaks powerfully to its cultural meaning as the most prominent surviving emblem of post-war social commitments to an inclusive, egalitarian and democratic citizenship.

## Footnotes

^1^ Harold Evans, ‘Harold Evans, Editor of the “*Sunday Times*” urges the need for a code of conduct in reporting racial matters’, *The Listener*, 16 July 1970, 75–8 at 75.

^2^ Evans, ‘Harold Evans’, 76.

^3^ Evans, ‘Harold Evans’, 77.

^4^ See Roberta Bivins, *Contagious Communities: Medicine, Migration and the NHS in Post War Britain* (Oxford, 2015).

^5^ See Ron Ramdin, *The Making of the Black Working Class in Britain* (Aldershot, 1987), George Kassimeris and Leonie Jackson, ‘Negotiating race and religion in the West Midlands: Narratives of inclusion and exclusion during the 1967–69 Wolverhampton bus workers’ turban dispute’, *Contemporary British History*, (2016), 1–23. DOI: 10.1080/13619462.2016.1226807 <http://www.tandfonline.com/loi/fcbh20> accessed 25 September 2016 and below, note 6. Racialized minorities working in public transport were depicted in photojournalism and editorial cartoons, but even here, the number of published images is dwarfed by images of ‘colour’ in the NHS.

^6^ See e.g., Paul Gilroy, *Ain’t No Black in the Union Jack: The Cultural Politics of Race and Nation* (Chicago, 1987, 1991); Paul B. Rich, *Race and Empire in British Politics* (2nd edn, Cambridge, 1990); Chris Waters, ‘“Dark Strangers” in our midst: Discourses of race and nation in Britain, 1947-1963’, *Journal of British Studies*, 36 (1997), 207–38, and most recently Kassimeris and Jackson, ‘Negotiating race and religion’. This focus on text and rhetoric has been particularly strong in the polemical historiography specifically addressing political and policy responses to ‘New Commonwealth’ migration, including Randall Hansen, *Citizenship and Immigration in Post-War Britain: The Institutional Origins of a Multicultural Nation* (Oxford, 2000) and Kathleen Paul, *Whitewashing Britain: Race and Citizenship in the Postwar Era* (Ithaca, 1997).

^7^ Pioneering work focused on imagery of imperial experiences and spaces: e.g., James Ryan, *Picturing Empire: Photography and the Visualisation of the British Empire* (Chicago, 1998). More recently, historians have turned the lens on Britain itself: e.g., Wendy Webster, *Englishness and Empire, 1939-1965* (Oxford, 2005); Catherine Hall and Sonya Rose, eds, *At Home with the Empire: Metropolitan Culture and the Imperial World* (Cambridge, 2006), and the excellent essays in Lee Grieveson and Colin MacCabe, eds, *Empire and Film* (Basingstoke, 2011) and Lee Grieveson and Colin MacCabe, eds, *Film and the End of Empire* (Basingstoke, 2011). On film and television representations of Britain’s Black and Asian populations, see Sarita Malik, *Representing Black Britain: Black and Asian Images on Television* (London, 2002), 1; Stephen Bourne, *Black in the British Frame: The Black Experience in British Film and Television* (London, 2001).

^8^ The principal exponents of this approach include Jordanna Bailkin, *The Afterlife of Empire* (Berkeley, 2012) and Camilla Schofield, *Enoch Powell and the Making of Postcolonial Britain* (Cambridge, 2013).

^9^ But see Bivins, *Contagious Communities* and other recent work; Anne MacClellan, ‘Victim or vector? tubercular Irish nurses in England 1930-1960’, in Catherine Cox and Hilary Marland, eds, *Migration, Health and Ethnicity in the Modern World* (Basingstoke, 2013) 104–25; Krista Maglen, ‘Importing trachoma: The introduction into Britain of American ideas of an “immigrant disease”, 1892-1906’, *Immigrants & Minorities,* 23 (2005), 80–99; John Welshman, ‘Tuberculosis and ethnicity in England and Wales, 1950-70’, *Sociology of Health & Illness*, 26 (2000), 858–82; John Welshman, ‘Tuberculosis, “race”, and migration, 1950-70’, *Medical Historian: Bulletin of Liverpool Medical History Society,* 15 (2003–4), 36–53; John Welshman, ‘Compulsion, localism, and pragmatism: The micro-politics of tuberculosis screening in the United Kingdom, 1950-1965’, *Social History of Medicine*, 19 (2006), 295–312. Gavin Schaffer, *Racial Science and British Society 1930-62* (Basingstoke and New York, 2008), does similar work in relation to British science.

^10^ The term ‘race’ today is generally used to describe the now-discredited concept of biologically distinctive subgroups of the human species, readily identifiable by phenotypic traits like skin pigmentation and hair texture but also distinguished by intrinsic and fixed differences in character and ability. ‘Race’ remains a variable of convenience in discussing the stratifying effects of hierarchies of human difference rooted in such beliefs. Although often used euphemistically for ‘race’, ‘ethnicity’ instead defines human subgroups on the basis of a (sometimes presumed) shared cultural heritage which may incorporate similarities in geographic or national origin, ancestry, language, ritual, cuisine, dress, and religion. Beyond the specialist literatures, ‘race’ was the dominant term used in post-war Britain to explain the presumed and perceived human differences between recently arrived populations (and sometimes their descendants) and the majority population. ‘Colour’ and ‘coloured’ were used by the majority population as socially acceptable generic terms in discussing population groups who could be visually distinguished by skin tone and hair texture. As the concept of biological race became increasingly untenable scientifically and politically, ‘race’ and ‘colour’ were gradually and partially replaced by ‘ethnicity’ as the preferred term for categorizing and discussing British populations. However, as discussed below, the content and subjects of such discussions remained largely the same: references either to ‘race’ or to ‘ethnicity’ equally evoked the culturally and visually distinctive non-white populations from Britain’s former colonies and Commonwealth, at least until the emergence of large-scale migration from Eastern Europe. In terms of their referents, ‘race’, ‘ethnicity’ and ‘colour’ were thus largely interchangeable in everyday discussions of im/migration and im/migrants during the period covered by this article, a near-equivalence which is visible in their use here.

^11^ Central Office of Information for the Ministry of Health, *The New National Health Service* (London, 1948), 1; see also Arthur Woodburn, *Your Health Service: How it Will Work in Scotland* (Edinburgh, 1948), 14, 27.

^12^ The term ‘equalitarianism’ was used explicitly to define the NHS in the contemporary press. Anonymous, ‘July 5*’, The Manchester Guardian* (5 July 1948), 4.

^13^ e.g., Imperial War Museum, London [IMW], Ministry of Works Official Collection, images MOW L 44149-60, L 44391 to 44395, L 49349, L 55636 to 55650, L 48839 to 48847. Images can be viewed online at < http://www.iwm.org.uk/collections> using the listed catalogue numbers.

^14^ Numerous historians and sociologists have documented the diversity of the British population in the twentieth century, particularly in London and other port cities, where so many of these images were produced. See the works above, n 5, n 7 and for a concise introduction to a growing field, Laura Tabili, ‘A homogenous society: Britain’s internal “others”, 1800–present’, in Hall and Rose, *At Home with the Empire*, 53–76.

^15^ See Randall Hansen, *Citizenship and Immigration in Post-War Britain* (Oxford, 2000), 17, 35–8.

^16^ Rich, *Race and Empire*, 188.

^17^ For a reflection on British attitudes towards this, Gavin Schaffer, ‘Re-Thinking the history of blame: Britain and minorities during the Second World War’, *National Identities*, 8 (2006), 401–19.

^18^ Hansen, *Citizenship and Immigration*, 3, 19.

^19^ Ian Gordon and Tony Travers, *Race, Immigration and Community Relations in Contemporary London* (London, 2006) <http://www.lse.ac.uk/geographyAndEnvironment/research/london/events/HEIF/HEIF1%202005-06/raceandimmigration/RaceAndImmigration.doc>.

^20^ See Wendy Webster, *Englishness and Empire*, 55–120 for post-war domestic visions of colonial subjects.

^21^ See e.g., the Pathé newsreel ‘Pakistan Nurses’, issued 26 December 1949, <http://www.britishpathe.com/video/pakistan-nurses/> accessed 19 July 2016. I have also found one reference to television coverage of a ‘colonial’ nurse training at London’s Middlesex Hospital then ‘returning home to disseminate her knowledge’ in c. 1954. Jan Stewer, ‘The Widdicomb File’, *Lancet*, 263 (1954), 1343–4, at 1344.

^22^ ‘On call to the nation’, BBC Television Service, 22 December 1958, <http://www.bbc.co.uk/archive/nhs/5145.shtml accessed 02/01/2016 at 53.07>.

^23^ See the National Archives, Kew [TNA] HO344/44, which documents precisely these planned efforts in 1959.

^24^ Malik, *Representing Black Britain*, 139; Bourne, *Black in the British Frame*, 182.

^25^ On official medical responses to post-war migration, see Bivins, *Contagious Communities*.

^26^ On relationships between empire and British responses to immigration and race, see especially Bill Schwarz, *The White Man’s World* (Oxford, 2011); Wendy Webster, *Imagining Home: Gender, ‘Race’ and National Identity* (London, 1998); Webster, *Englishness and Empire*.

^27^ e.g., *Parliamentary Debates* (Commons), 463, 8 April 1949, 2439–48; *Parliamentary Debates* (Lords), 163, 28 June 1949, 345–8; *Parliamentary Debates* (Commons), 468, 19 October 1949, 629–45; and in the press, ‘Parliament’, *The Times,* 9 April 1949, 2; ‘Amending the Health Act’, *The Times*, 14 May 1949, 5; ‘Wigs for visitors’, *The Times*, 21 May 1949, 5.

^28^ For instance, Howard Markel and Alexandra Minna Stern, ‘The foreignness of germs: The persistent association of immigrants and disease in American society’, *The Milbank Quarterly*, 80 (2002), 757–88. Case studies and examples include Alison Bashford, *Imperial Hygiene: A Critical History of Colonialism, Nationalism and Public Health* (Basingstoke, 2003); Alan Kraut, *Silent Travelers: Germs, Genes and the Immigrant Menace* (Baltimore, 1994); Lara Marks and Michael Worboys, eds, *Migrants, Minorities and Health: Historical and Contemporary Studies* (London, 1997); Nayan Shah, *Contagious Divides: Epidemics and Race in San Francisco’s Chinatown* (Berkeley, 2001). For reflections in relation to contemporary concerns: Charles T. Adeyanju and Nicole Neverson, ‘“There will be a next time”: Media Discourse about an “apocalyptic” vision of immigration, racial diversity, and health risks’, *Canadian Ethnic Studies*, 39 (2007), 79–105; Nick King, ‘Security, disease, commerce: Ideologies of post-colonial global health’, *Social Studies of Science*, 32 (2002), 763–89; Nancy Tomes, ‘Public health then and now: The making of a germ panic, then and now’, *American Journal of Public Health*, 90 (2000), 191–8.

^29^ See Mica Nava, ‘Sometimes antagonistic, sometimes ardently sympathetic: Contradictory responses to migrants in postwar Britain’, *Ethnicities*, 14 (2014), 458–80; Bailkin, *Afterlife of Empire*.

^30^ Anthony Richmond, *The Colour Problem: A Study of Racial Relations* (London, 1955), 29.

^31^ Sheila Patterson, *Dark Strangers: A Sociological Study of the Absorption of a Recent West Indian Migrant Group in Brixton, South London* (London, 1963), 230; Ruth Glass, *Newcomers: The West Indians in London* (London, 1960), 3.

^32^ Modern Record Centre, Coventry [MRC], MSS.292/805.7/3/286; Leslie Stephens, ‘Draft Report: employment of coloured workers in the Birmingham area. The report of an enquiry initiated by the Birmingham Christian social council and undertaken by the Race Relations Group of Fircroft College, Birmingham. Private and confidential—not for publication’, 1956, 3.

^33^ Stephens, ‘Draft Report’, 9.

^34^ MRC, MSS.292/805.7/3/48, Dr G. Bertram, *The Times*, 30 September 1958, quoted in Institute of Race Relations [IRR], *Coloured People in Great Britain Summary of Press News and Comment, September 1958* (London, 1958), 12.

^35^ See e.g., Jeremy Seabrook, ‘Portrait of Blackburn’, *The Listener*, 27 (1970), 267–71, at 270; Albert Maria Gomes, ‘I am an immigrant’, *The Listener*, 3 (1968), 425–6.

^36^ Brigadier Sir John Smyth, *Parliamentary Debates* (Commons), 17 February 1961, 634, 1929-2024 at 1952.

^37^ On the use of ‘ethnicity’ as a substitute for ‘race’, see J. S. Huxley and A. C. Haddon, *We Europeans: A Survey of ‘Racial’ Problems* (London, 1935), 107, 108, 164–5, often credited with introducing the modern use of ‘ethnicity’ as a biosocial category; Guido Bolaffi, Raffaele Bracalenti, Peter Braham and Sandro Gindro, eds, *Dictionary of Race Ethnicity and Culture* (London, 2003), 96–102 and contrast with David Kelleher, ‘A defence of the uses of the terms “ethnicity” and “culture”‘, in David Kelleher and S. M. Hillier, *Researching Cultural Differences in Health* (Abingdon, 2002), 69–90.

^38^ Peter Salisbury, ‘Giles’s Cold War: How Fleet Street’s Favourite Cartoonist Saw the Conflict’, *Media History*, 12 (2006), 157–75. Near-contemporary analyses of the power of cartoons in shaping and reflecting social opinion include David Langdon, ‘Satire and the artist’, *Journal of the Royal Society of Arts*, 114 (1966), 760–78; Thomas Milton Kemnitz, ‘The cartoon as a historical source’, *Journal of Interdisciplinary History*, 4 (1973), 81–93. More recent debates on morality and humour are summarized in E. M. Dadlez, ‘Truly funny: Humour, irony and satire as moral criticism’, *Journal of Aesthetic Education*, 45 (2011), 1–17.

^39^ Adrian W. B. Randolph, ‘Preface’ in Angela Rosenthal, ed., with David Bindman and Adrian W. B. Randolph, *No Laughing Matter: Visual Humor in Ideas of Race, Nationality and Ethnicity* (Hanover, NH, 2016), ix–xii.

^40^ Langdon, ‘Satire and the artist’, 767–8. Gavin Schaffer has recently analysed similar trends in Johnny Speight’s BBC sitcom ‘Till Death Us Do Part’, noting that while the audience may have been invited to laugh at racist Alf Garnett, they were also allowed to identify with him. Gavin Schaffer, ‘“Till Death Us Do Part” and the BBC: Racial politics and the British working classes 1965-75’, *Journal of Contemporary History*, 45 (2010), 454–77.

^41^ Stuart Hall, ‘The whites of their eyes: racist ideologies and the media’, in G. Bridges and R. Brunt, eds, *Silver Linings: Some Strategies for the Eighties* (London, 1981), 28–52 at 43, quoted in Schaffer, ‘Till Death Us Do Part’, 458.

^42^ See Bivins, *Contagious Communities*, 62–109, 168–226; Welshman, ‘Compulsion, localism, and pragmatism’; Welshman, ‘Tuberculosis, “race”, and migration’; Ian Convery, John Welshman, and Alison Bashford, ‘Where is the border? Screening for tuberculosis in the United Kingdom and Australia, 1950-2000’, in Alison Bashford, ed., *Medicine at the Border: Disease, Globalization and Security, 1850 to the Present* (Basingstoke, 2006), 97–115.

^43^ See e.g., the description of ‘immigrants’ as ‘easily recognized’ (implicitly by skin colour) and TB incidence charts presented in ‘Tuberculosis among immigrants in Britain: Memorandum from the Joint Tuberculosis Committee’, *British Medical Journal*, 1 (1978), 1038–40, at 1039.

^44^ General practice was poorly represented in the visual culture of NHS, particularly after its initial launch, and few press photographs documenting non-white patients or practitioners in general practice were published, e.g. Thurston Hopkins’ images of a London-based South Asian GP and his patient, photographed for, but unpublished by the *Picture Post* in 1955 (Getty Images 146420904, 146420910, 146420911, viewable online at < http://www.gettyimages.co.uk/>).

^45^ J. Brown, ‘Immigrant loophole’, *Daily Mail*, 19 February 1965, 9.

^46^ ‘The day Abdul was made an Englishman’, *Daily Mail*, 19 February 1965, 9.

^47^ On the x-ray scheme, see TNA, MH 148/28, MH148/29, MH148/30.

^48^ As e.g. in John Tenniel, ‘Back!’, *Punch*, 9 October 1892.

^49^ The pre-2010 cartoons also included one depicting three infant African states, and another featuring boxer Muhammad Ali. Two *Punch* images also used a hospital setting to convey political claims.

^50^ The British Cartoon Archive, University of Kent at < http://www.kent.ac.uk/cartoons/ > [BCA], catalogue record [CR] 25058. Keith Waite, ‘The world of Keith Waite’, *Daily Mirror*, 11 September 1973.

^51^ See Hansen, *Citizenship and Immigration*, 111.

^52^ BCA, catalogue reference MC1043. Michael Cummings, ‘So it’s alright to let *humans* through—*they* may only have smallpox!’, *Daily Express*, 17 January 1962.

^53^ On the smallpox outbreaks see Bivins, *Contagious Communities*, 115–67.

^54^ IRR, ‘Coloured people in Britain’, 3, citing coverage in *The Tablet*, 13 September 1958, and in *West Africa*, 6 September 1958, respectively.

^55^ See e.g. the images of doctors, nurses and hospital wards, including a number documenting Aneurin Bevan’s visits to NHS sites, held by the Getty Picture Archives, <http://www.gettyimages.co.uk/editorialimages/archival> accessed 20 February 2016.

^56^ ‘These coloured nurses are happy’, *Daily Herald*, 3 March 1955, 3.

^57^ ‘Life would be harder for all of us without coloured labour’, *The Times*, 27 January 1965, 6.

^58^ ‘In memory of Nurse Edith Cavell, *The Times*, 13 October 1965, 20; ‘The Duchess of Kent presents awards to nurses’, *The Times*, 29 September 1967, 14; ‘Computer course at Westminster’, *The Times*, 18 October, 1965, 22; ‘Rehearsing medieval jousting for the 1066 pageant’, *The Times*, 10 October 1966, 18; ‘Picture gallery’, *The Times*, 18 October 1971, 1.

^59^ ‘Flu threatens the hospital service’, *The Times*, 17 December 1969, 14.

^60^ T. Smith, ‘Verdict on the NHS: could do better’, *The Times,* 5 July 1978, 16.

^61^ H. Kretzmer, ‘Is the NHS fit to keep us all healthy?’, *Daily Mail*, 14 October 1986, 29

^62^ Jo-Ann Goodwin, ‘How the NHS betrayed my mum’, *Daily Mail,* 24 January 1998, 12–13.

^63^ All of the editorial images to which I refer in this article can be viewed at the British Cartoon Archive’s website, which is searchable both by key terms and, using the ‘advanced search’ option, by the catalogue reference numbers in each note.

^64^ Salisbury, ‘Giles’s Cold War’, 158.

^65^ BCA, catalogue reference GAN/412, Giles (Ronald Carl Giles), ‘Now there’s an embarrassment for yer, Tosh’, *Sunday Express*, 7 September 1958.

^66^ e.g. BCA, catalogue reference 12950, Jak, ‘Hang on Abdul, it says shrunken glands, not Duncan Sandys’, London *Evening Standard*, 23 February, 1968; BCA, catalogue reference 13270, Jon (William John Philpin Jones), ‘You see, doc, I dropped my “I-Back-Enoch” placard on my foot’, *Daily Mail*, 24 April 1968.

^67^ BCA, catalogue reference 06296, Trog (Wally Fawkes), uncaptioned cartoon, *New Statesman*, 20 November 1964.

^68^ ‘Do the immigrants pay their way?’, *Illustrated London News*, 18 May 1968. Unlike the very detailed information available for doctors, data on the ethnicity of NHS nurses in this period is partial and patchy; training numbers are the best available proxy.

^69^ With thanks to Jack Saunders for this observation. Figure 3: BCA, catalogue reference 19769, Giles ‘He’s a bit niggly because I confiscated a bottle of Scotch he’d smuggled under the sheets’, *Daily Express*, 25 February 1971.

^70^ See e.g., BCA, catalogue reference 46078, Mac (Stan McMurtry), *Daily Mail*, 20 August 1985, BCA, catalogue reference 46073, Jak (Raymond Jackson), London *Evening Standard*, 24 November 1987.

^71^ BCA, catalogue reference 06413, Jak, ‘Don’t just stand there—they’re wanted at Sotheby’s’, London *Evening Standard*, 03 December 1964. His NHS related cartoons routinely included a multi-ethnic nursing force throughout his career. See e.g. BCA, catalogue reference 44878, Jak, London *Evening Standard,* 20 September 1985, and numerous others.

^72^ BCA, catalogue reference 98177, Mac, ‘Blow no whistles, see no whistles, hear no whistles’, *Daily Mail*, 19 February 2013. See also BCA, catalogue reference 99719, Mac, ‘Before we start, put out of your mind any rubbish you may have heard about high-risk hospitals’, *Daily Mail,* 25 October 2013. Both pieces are characteristic of the darker tones in which NHS medical staff were portrayed during this episode.

^73^ BCA, catalogue reference 16646, Stanley Franklin, ‘Is this some of the “alien–occupied territory’ Powell’s talking about?”‘, *Daily Mirror*, 13 November 1969.

^74^ David Langdon, ‘I beg you to excuse the release of any deep-seated prejudices while I’m going under’, *Punch*, 1969.

^75^ Chris Dunkley, ‘Immigrant doctors criticized’, *The Times*, 19 February 1969, 1. Like earlier stories about nurses, this *Times* account again defends the NHS from any implications of racism: ‘There was, the consultant said, no suggestion of a colour problem. It was a question of doctors from any foreign country’.

^76^ BCA, catalogue reference 14854, Jak, ‘You’re sure he speaks English? I only came in for my tonsils!’, London *Evening Standard*, 21 February 1969.

^77^ BCA, catalogue reference MW2517, Emmwood (John Musgrave-Wood), ‘More Saturday sonnets’, *Daily Mail*, 22 February 1969.

^78^ BCA, catalogue reference 14856, David Meyers, ‘Our immigrant doctors I find splendid—but I am a bit worried by Jekyll’, London, *Evening Standard*, 21 February 1969.

^79^ BCA, catalogue reference 14867, Michael Cummings, ‘He doesn’t speak English but what’s worse, he’s curing all the patients I haven’t been able to cure…’ *Daily Express*, 21 February 1969, and BCA, catalogue reference 14868, Trog, *Daily Mail*, 21 February 1969. Of course the witchdoctor trope was not novel. On the cinematic use of the witchdoctor as a marker of enduring African primitivism and fragile African modernity, see Wendy Webster, ‘Mumbo-jumbo, magic and modernity: Africa in British cinema 1946-65’, in Grieveson and MacCabe, *Film and the End of Empire*, 237–50; Philip Zachernuk, ‘Who needs a witch-doctor? Refiguring British colonial cinema in the 1940s’, in Grieveson and MacCabe, *Film and the End of Empire*, 95–118.

^80^ BCA, catalogue reference 05862, Stanley Franklin, ‘Emergency in the wards: doctor shortage hits hospitals’, *Daily Mirror*, 30 July 1964. Captioned ‘I’m sick of this new ‘Temporary’—where are we going to get frog legs at this time of night?’, it depicted two nurses complaining in the background as a masked witch doctor performed at the bedside of an anxious looking patient.

^81^ BCA, catalogue reference 19708, Mac, *Daily Sketch*, 17 February 1971.

^82^ Quoted in ‘Biography: Stan McMurtry [Mac]’, BCA <https://www.cartoons.ac.uk/artists/stanmcmurtry/biography> accessed 4 January 2016.

^83^ BCA, catalogue reference 17886, Jak, ‘Take the sheets off O’Reilly, He’s only got double pneumonia!’ London *Evening Standard,* 29 May 1970; BCA, catalogue reference CG/1/1/1/2253, Giles, ‘Hurt his toe kicking a policeman—what’s the matter my little angel, too hot?’, *Daily Express,* 28 August 1973; BCA, catalogue reference CG/1/4/2/1/31/18, Giles, ‘He says his case is urgent. He’s been feeling a bit depressed since 29 September1905’, *Daily Express*, 20 October 1975. This image shows an ethnically mixed group of junior doctors of both sexes relaxing in the explicitly NHS Junior Doctor’s Rest Room, a response to the then ongoing junior doctors’ strike; BCA, catalogue reference 31920, Giles, ‘“Charge Nurse was demoted this week for less than asking a patient if she could have his guts for garters, Nurse’, *Daily Express*, 22 March 1978.

^84^ See e.g. the testimony of Nigerian nurse Elsie George in Webster, *Imagining Home*, 103–4; Sharon Beishon, Satnam Virdee, Ann Hagell, *Nursing in a Multi-Ethnic NHS* (London, 1995).

^85^ ‘Do the immigrants pay their way?’, *Illustrated London News*; the article also cites numbers and includes an image of London Transport workers. See Julian M Simpson, Aneez Esmail, Virinder S Kalra and Stephanie J Snow, ‘Writing migrants back into NHS history: Addressing a “collective amnesia” and its policy implications’, *Journal of the Royal Society of Medicine*, 103 (2010), 392–6. There were also large numbers of ethnic minority general practitioners, but general practice rarely attracted broadcast or cinematic coverage. Julian Simpson, ‘Reframing NHS history: Visual sources in a study of UK-based migrant doctors’, *Oral History*, *42* (2014), 56–68.

^86^ J. Burke, ‘Something for nothing: a birthday celebration’, BBC 1, 27 June 1968. <http://www.bbc.co.uk/archive/nhs/5157.shtml at 07.14-07.34> accessed 1 February 2016.

^87^ Burke, ‘Something for Nothing’. The Asian patients are momentarily visible at 02.25 and 02.31.

^88^ Burke, ‘Something for Nothing’, at 29.17-29.25.

^89^ Virginia Ironside, ‘TV’, *Daily Mail*, 28 June 1968, 3.

^90^ Seabrook, ‘Portrait of Blackburn’, 270.

^91^ On race relations in British sitcoms, see Gavin Schaffer, ‘Till Death Us Do Part’.

